# Recent advances in understanding myelofibrosis and essential thrombocythemia

**DOI:** 10.12688/f1000research.8081.1

**Published:** 2016-04-19

**Authors:** William Vainchenker, Stefan N. Constantinescu, Isabelle Plo

**Affiliations:** 1 Gustave Roussy, Paris, France; 2 Universite Paris-Saclay, Gustave Roussy, Paris, France; 3 Signal Transduction & Molecular Hematology Unit, Ludwig Institute for Cancer Research, Brussels, Belgium; 4 de Duve Institute, Université catholique de Louvain, Brussels, Belgium

**Keywords:** myelofibrosis, thrombocythemia, Myeloprolifarative neoplasms

## Abstract

The classic *BCR-ABL*-negative myeloproliferative neoplasms (MPNs), a form of chronic malignant hemopathies, have been classified into polycythemia vera (PV), essential thrombocythemia (ET), and primary myelofibrosis (PMF). ET and PMF are two similar disorders in their pathogenesis, which is marked by a key role of the megakaryocyte (MK) lineage. Whereas ET is characterized by MK proliferation, PMF is also associated with aberrant MK differentiation (myelodysplasia), leading to the release of cytokines in the marrow environment, which causes the development of myelofibrosis. Thus, PMF is associated with both myeloproliferation and different levels of myelodysplastic features. MPNs are mostly driven by mutated genes called MPN drivers, which abnormally activate the cytokine receptor/JAK2 pathway and their downstream effectors. The recent discovery of *CALR* mutations has closed a gap in our knowledge and has shown that this mutated endoplasmic reticulum chaperone activates the thrombopoietin receptor MPL and JAK2. These genetic studies have shown that there are two main types of MPNs: JAK2V617F-MPNs, including ET, PV, and PMF, and the MPL-/CALR-MPNs, which include only ET and PMF. These MPN driver mutations are associated with additional mutations in genes involved in epigenetics, splicing, and signaling, which can precede or follow the acquisition of MPN driver mutations. They are involved in clonal expansion or phenotypic changes or both, leading to myelofibrosis or leukemic transformation or both. Only a few patients with ET exhibit mutations in non-MPN drivers, whereas the great majority of patients with PMF harbor one or several mutations in these genes. However, the entire pathogenesis of ET and PMF may also depend on other factors, such as the patient’s constitutional genetics, the bone marrow microenvironment, the inflammatory response, and age. Recent advances allowed a better stratification of these diseases and new therapeutic approaches with the development of JAK2 inhibitors.

## Introduction

Myeloproliferative disorders are characterized by excess proliferation of progenitors belonging to the myeloid lineages (myeloproliferation), leading to an excess of mature functional blood cells ^1^. They are all clonal disorders of the hematopoietic system deriving from the transformation of a hematopoietic stem cell (HSC). Among the spectrum of myeloid malignancies they lie at one extreme, characterized only in principle by myeloproliferation (without differentiation defects), in contrast to myelodysplastic syndrome (MDS) (predominant differentiation defects) and acute myeloid leukemia (AML) (blockage in differentiation). The classic *BCR-ABL*-negative myeloproliferative neoplasms (MPNs) have been classified into three entities: polycythemia vera (PV), essential thrombocythemia (ET), and primary myelofibrosis (PMF). These diseases have common complications: thrombosis or, more rarely, hemorrhages and leukemic transformation. ET is essentially a disorder of the megakaryocyte (MK) lineage with an excess platelet production ^2^. PMF is defined by the presence of bone marrow fibrosis (excess of collagen fibers) ^3^. This is also mainly a disorder of the MK/platelet lineage but is also associated with granulocytic proliferation. The typical forms of PV, ET, and PMF are quite different clinically and have different prognosis. ET and PV can progress to secondary myelofibrosis. Certain ET cases are associated with an erythroid hyperplasia and can progress to a true PV or may remain a form “fruste” of PV. Furthermore, boundaries between ET and PMF are not well standardized. A fourth entity has been described, pre-PMF (or early PMF or prefibrotic myelofibrosis), which corresponds to an ET with a high probability of progression to myelofibrosis and a worse prognosis than classic ET ^4^.

The molecular pathogenesis of *BCR-ABL*-negative MPNs is now in large part understood because of recent advances in sequencing techniques, particularly with results derived from next-generation sequencing (NGS) techniques. Recently, the discovery of mutations in the calreticulin ( *CALR*) genes has closed a gap in the knowledge of the physiopathogenesis of these disorders, particularly for ET and myelofibrosis.

In this review, we will focus on the molecular pathogenesis of MPNs, particularly of ET and PMF. However, somatic acquired mutations cannot summarize the entire pathogenesis of these disorders and other factors such as the constitutional genetics, the bone marrow niche environment, the cytokine release, and the inflammatory response, as well as aging, play important roles in the heterogeneity of these disorders.

## Discovery of the mutations in exon 9 of the *CALR* gene in ET and PMF reinforces the hypothesis that *BCR-ABL*-negative MPNs are driven by an abnormal activation of JAK2

In 2005, a major advance in the understanding of the pathogenesis was the discovery of the somatic acquired recurrent mutation *JAK2*V617F, which is associated with more than 70% of MPNs; namely 95% of PV, 50% of ET, and 60% of PMF ^5– 8^. The V617F mutation is located in the pseudokinase domain of JAK2. The V617F mutation appears to prevent the physiologic inhibition and also to directly activate the kinase domain of JAK2 ^9^. JAK2V617F gain-of-function and the constitutive signaling at sufficient expression levels require cytokine receptors, particularly homodimeric type I receptors. The identification of the *JAK2*V617F mutation has been a cutting-edge discovery in the pathogenesis of MPNs. This has led to the implication of the cytokine receptor/JAK2/STAT5 signaling pathway in their pathologies and the subsequent discovery of other recurrent mutations in this pathway, such as *JAK2* exon 12 in 2% of PV ^10^, and activating mutations in the thrombopoietin receptor *MPL.* These mutations located in exon 10 of *MPL* target the W515 residue, which plays a central role in preventing spontaneous activation of the receptor ^11^. When W515 is substituted by 17 other amino acids—most frequently, Leu and Lys—TpoR/MPL becomes constitutively active and oncogenic ^12^. These mutations are found only in ET and PMF, with frequencies of approximately 3% and 5–8% in ET and PMF, respectively ^13^. The somatic *MPL*S505N is a rare recurrent sporadic mutation in ET and PMF that in certain familial thrombocytosis cases is found in the germline ^14^. Finally, very rare somatic mutations in *LNK*, a negative regulator of JAK2 kinase activity, have been described in ET and PMF ^15^. *JAK2*V617F and *MPL* mutations are only very rarely found in the same patient sample and when both are present they are most of the time in different cells, suggesting that they belong to different clones or subclones.

**Figure 1.**
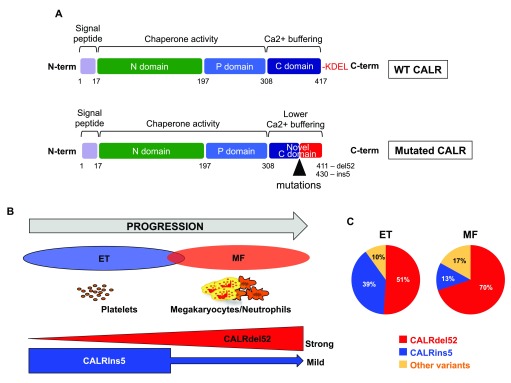
Calreticulin (CALR) and CALR mutation in essential thrombocythemia (ET) and myelofibrosis (MF). ( **a**) CALR protein structure. CALR includes different domains responsible for the two major activities (chaperone and calcium buffering). The mutations lead to altered C-terminal part with loss of KDEL (retrieval and retention domain in endoplasmic reticulum) and generation of a new tail with low calcium-buffering activity. ( **b**) Progression from ET to MF with CALR mutants. *CALR*del52 induces ET always progressing to MF in mice in contrast to *CALR*ins5. Thus, *in vivo* modeling of CALRdel52-induced pathologic effects induces a disorder characterized by a continuum between ET and MF. ( **c**) Pie chart of the different *CALR* mutations in patients with ET and MF.

In 2013, it was evident that 55% of ET and 65–70% of PMF cases were linked to *JAK2*V617F and *MPL* exon 10 mutations. Activation of the cytokine receptor/JAK2 pathway was a common feature. In approximately 40% of ET and PMF, there were no recurrent mutations in genes involved in signaling. At the end of 2013, the teams of Kralovics ^16^ and Green ^17^ discovered mutations (indel) in the *CALR* gene in 25–30% of ET and PMF that were negative for *JAK2* and *MPL* mutations. More than 50 mutations have been described, but all are in exon 9 and induce a +1 (−1+2) frameshift, leading to a new C-terminal peptide and the absence of the KDEL sequence, a retention sequence for the endoplasmic reticulum (ER) ( Figure 1). The C-terminus is almost identical among mutations with about 30 common amino acids. These new sequences completely change the charge of the molecule. The most frequent mutation, del52 (55% of the mutations), also called type 1, eliminates almost all the negative charges, whereas the ins5 (30%)—also called type 2—eliminates about half of these charges. According to these changes, the other mutations have been classified as type 1- or type 2-like. Physiologically, CALR is not a signaling molecule but an ER chaperone involved in the quality control of N-glycosylated protein and in calcium storage in the ER ^18^. However, the fact that the *CALR* mutations were also mutually exclusive with *JAK2*V617F and *MPL* mutations in ET and PMF, together with preliminary results showing that del52 mutations could activate STAT5, suggested that the CALR mutants were involved in signaling ^16^. Recent studies have largely reinforced this hypothesis by showing that CALR mutants activate the MPL receptor after binding to its N-glycosylated residues in the ER ^19, 20^. This activation required the positive charge of the C-terminus peptide, the lectin binding domain, and the extracellular N-linked sugars of MPL. There is evidence that the CALR mutant associated with MPL traffics to the cell surface in an immature N-glycosylated form ^19^. In this case, MPL activation can occur anywhere from the ER to the cell surface. Moreover, CALR mutants are secreted proteins, which may be able to activate other cells, especially monocytes, to secrete inflammatory cytokines ^21^. CALR mutants are not able to activate other cytokine receptors different from MPL—except granulocyte colony-stimulating factor receptor (G-CSF-R). However, this activation is weak and does not allow the autonomous growth of factor-dependent cell lines.

Thus, it appears that there are two main types of *BCR-ABL*-negative MPNs. The first one is the *JAK2*V617F MPNs (∼70% of MPNs), which includes three disorders: ET, PV, and PMF; the second one consists of the *CALR* and *MPL* mutated MPNs (20% of MPNs), which usually includes only ET and PMF although *CALR* mutations have been described in very rare cases of PV associated with a thrombocytosis. The remaining MPNs are called triple-negative (10%). These appear to be heterogeneous disorders, but a large fraction are associated with increased JAK/STAT signaling ^22^. Certain triple-negative MPNs are related to atypical *MPL* or *JAK2* mutations ^23, 24^. A fraction of the so-called triple-negative ET might not be MPNs, but polyclonal disorders, such as hereditary thrombocytosis with germline mutations. Furthermore, the triple-negative PMF, which is of poor prognosis, may not be bona fide MPNs, but more a myelodysplastic syndrome associated with myelofibrosis ^25^. This underscores the difficulties for classifying myeloid hematological malignancies, which might represent a spectrum of diseases with proliferation and differentiation defects at different levels.

### Signaling mutations drive the MPN phenotype

One way to demonstrate that these mutations are really the MPN drivers is to create mouse models. Mutations in *JAK2*V617F, *MPL*, and *CALR* are capable of reproducing the MPN phenotype(s) in mice. *JAK2*V617F induces a myeloproliferative disorder—usually PV but also ET in some models ^26– 29^—which may progress to myelofibrosis. The unique models that have been presently described so far for *MPL*W515 and *CALR* mutations are bone marrow transplantations after retroviral transfer. In both cases the mice develop thrombocytosis, which progresses to myelofibrosis—quickly in the case of MPLW515L/A and more slowly for CALRdel52 ^11, 20^.

Importantly, in all these models, mice do not develop true PMF, but a secondary myelofibrosis (post-PV or post-ET). Thus, myelofibrosis can be the natural evolution of ET, without requiring other additional genetic abnormalities. Similarly high TPO levels in mice can induce a very severe myelofibrosis ^30, 31^. Overall, an exaggerated stimulation of the MK lineage can lead to myelofibrosis. These results could suggest that PMF may require other events (genetic/environmental).

One major limitation of the present mouse models is that the disease originates from several hematopoietic stem cells, while human MPNs exhibit a clonal hematopoiesis originating from a single hematopoietic stem cell ^32^. Therefore, the first part of the human disease (how a single mutated HSC becomes predominant) is not studied in these models ^32^. Other factors, including oncogenic cooperation, may be necessary for clonal dominance (see below).

### Subtle changes in the activation mechanisms of JAK2 among mutants may partially explain the different phenotypes of the MPNs

Although the different mutations induce the activation of JAK2, they do not lead to the same phenotype. For example, JAK2V617F can be associated with ET and PV, inducing hyperplasia of either MK or erythroid cells, depending on the conditions. One determining factor is clearly the number of *JAK2*V617F gene copies (heterozygous versus homozygous mutation) ^28, 33^. However, this is just one factor in determining MPN heterogeneity.

Another example is the *CALR-*mutated and *JAK2*V617F ET, which display different clinical and biological features, although in both cases the disease is related to the activation of the MPL/JAK2 pathway ^20, 34^. One obvious difference is related to the fact that JAK2V617F—in contrast to CALR—activates not only the MK cell line but also the erythroid and granulocytic lineages, explaining differences in the hematocrit and polymorphonuclear count. However, among the most marked differences are the higher level of thrombocytosis and the decreased frequency of thrombotic events in the *CALR* mutated ET ^35, 36^. The most striking difference concerns the allele frequency of the mutation: in ET, the *JAK2*V617F variant allele frequency is approximately 15% in granulocytes (30% of mutated cells) but is approximately 40% or more for mutated *CALR* (80% of the cells) ^37^. Therefore, a greater clonal advantage at the level of HSCs is conferred by mutated *CALR* versus *JAK2*V617F, even if both diseases are dependent on MPL. Subtle differences in signaling pathways downstream of MPL/JAK2 might also be involved. For example, CALR mutants moderately activate the PI3K/AKT pathway, and PI3K inhibitors are not able to synergize with JAK2 inhibitors, contrasting what was observed for JAK2V617F ^19, 38, 39^. The type of activated STAT could also play a role since MPL/JAK2 can activate STAT1, 2, 3, and 5, which may have markedly different effects on HSC and MK biology ^40– 43^.

Furthermore, among *CALR* mutated ET, *CALR*del52 and *CALR*ins5 may define two different subtypes of diseases characterized by different levels of thrombocytosis and evolution. *CALR*del52 ET can progress to secondary myelofibrosis much more frequently than *CALR*ins5 ET ^44^, with an important predominance of *CALR*del52 in PMF ( Figure 1). In the mouse models, *CALR*del52-induced thrombocytosis progresses to myelofibrosis, but this progression is rarely observed for *CALR*ins5.

Again, such differences might reflect subtle differences in the activation of the MPL/JAK2 pathway or activation of new signaling pathways. Indeed, the CALRdel52 has nearly lost all its capacity to bind calcium in its C-terminal domain (low affinity, high capacity) in contrast to CALRins5. This may lead to a leak of calcium from the ER to the cytoplasm and a different signaling in MKs and in HSCs ^44^.

## The somatic landscape of acquired mutations demonstrates that additional somatic mutations are present in MPNs but predominantly in PMF

Early studies on *JAK2*V617F MPNs have suggested that, in certain cases, *JAK2*V617F is not the initiating event but that it could be preceded by other mutations. With genome-wide approaches, it could be shown that some patients have *TET2* mutations (∼15%) ^45^. Subsequently, mutations in *ASXL1* mutations (10–15%) were found in PMF ^46^.

With the development of whole exome sequencing, it could be demonstrated that mutations in epigenetic regulators (such as *TET2*, *DNMT3A*, *ASXL1*, *EZH2*, and *IDH1/IDH2*) and in spliceosome components (such as *SRSF2*, *U2AF1*, and *SF3B1*) were present in *BCR-ABL*-negative MPNs harboring *JAK2*/ *MPL*/ *CALR* mutations ^17, 47, 48^. Other mutations were also directly associated with leukemic progression, such as *p53*, *RUNX1*, *CBL*, and deletion in *IKAROS* ^49– 51^. These mutations can be associated, and the most frequent co-mutations concern *SRSF2* associated with *TET2* or *ASXL1* or *IDH* ^52^.

### The additional mutations are mainly phenotypic modifiers discriminating between ET and PMF

In contrast to mutations in signaling genes (MPN driver genes), which are rare in other myeloid malignancies, the additional mutations are not specific to MPNs and are found with a higher frequency in MDS and in mixed MDS/MPN disorders, such as chronic myelomonocytic leukemia ^53, 54^.

Biological studies and mouse models showed that they may cooperate with MPN drivers to favor clonal dominance ( *TET2* or *DNMT3A*), to modify disease phenotype, or to promote either progression to myelofibrosis or leukemic transformation ( *ASXL1*, *IDH1/2*, *EZH2*, and *TP53*).

Clonal dominance genes, such as *TET2* or *DNMT3A*, are associated with all types of MPNs with low difference in frequency (∼12% in ET and 18% in PMF). However, all the other mutations are almost exclusively found in PMF ^17, 55^. In more than 80% of PMF, mutations of epigenetic regulators or spliceosome components are found, but they are identified in less than 25% of ET. Furthermore, in approximately 50% of PMF, two or more of these non-‘MPN driver’ genes are co-mutated. Moreover, *CALR* is the first mutation in nearly all cases and additional mutations are secondary in disease evolution ^16, 17, 47^. In contrast, *JAK2*V617F can be preceded by mutations such as in *TET2*, *DNMT3A*, and *ASXL1*, whereas the inverse can be also observed.

**Figure 2.**
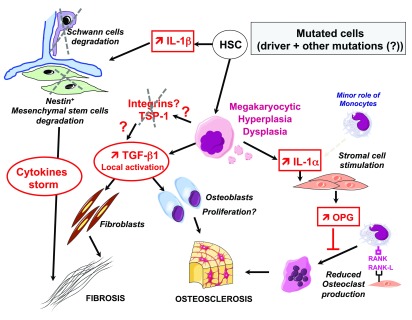
Role of microenvironment in the development of myelofibrosis Mutated dysplastic megakaryocytes (MKs) are responsible for the myelofibrosis and osteoclerosis by inducing the release of (i) non-activated transforming growth factor β1 (TGFβ1), which is activated in the bone marrow environment by a so far uncharacterized mechanism, possibly via integrins and matrix such as fibronectin and thrombospondin (TSP). Fibrosis begins around MKs associated with the proliferation of fibroblasts and eventually osteoblasts, (ii) interleukin-1α (IL-1α) is released and induces osteoprotegerin (OPG) by t stromal cells, a decoy receptor that blocks osteoclast production. Mutated hematopoietic stem cells (HSCs) induce the increase in IL-1α and the subsequent degradation of Schwann cells and mesenchymal stem cells, leading to fibrosis and osteosclerosis through cytokine storm and providing a favorable environment for the hematopoietic clone.

**Figure 3.**
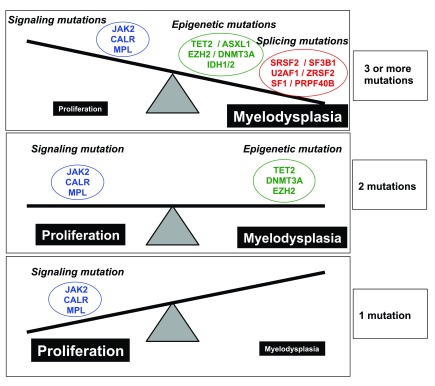
The type and the number of mutations determine the phenotype of the disease Boundaries between diseases are not easy to determine and could be dependent on the types or the number of mutations. Proliferation is driven mainly by signaling mutations ( *JAK2*, *CALR*, and *MPL*) while most of the mutations in epigenetic regulators and spliceosome components lead to differentiation defects. Thus, it can be considered that primary myelofibrosis (PMF) is not a pure myeloproliferative neoplasm (MPN) but a disorder with both myeloproliferative and myelodysplastic components. The heterogeneity of the disease and its prognosis are dependent on the respective levels of each component, and prognosis is poor if myelodysplastic features are predominant.

Two non-mutually exclusive explanations can be invoked: (1) *CALR* mutations have a much higher capacity to provide clonal dominance than *JAK2*V617F and may not require other associated genetic events for disease initiation. (2) *JAK2*V617F gives rise to MPNs, which occur approximately 10 years later than *CALR* mutated MPNs. The genes, which precede *JAK2*V617F occurrence, are associated with age-related clonal hematopoiesis ^56, 57^, *JAK2*V617F MPNs being secondary to aging. Indeed, the order of acquisition of mutations is important in the phenotype of the disease, particularly for *TET2* and *DNMT3A* ^58, 59^. Moreover, when the *JAK2*V617F mutation is acquired on an age-related hematopoiesis, leukemia or myelodysplastic syndrome transformation may occur on the initial *JAK2*V617F-negative clone ^60, 61^. The fact that the number of acquired mutations allows a good discrimination between ET (one mutation in the MPN driver gene plus eventually another driver mutation) and PMF (one mutation in the MPN driver gene and mutations in one or several other driver genes) is in agreement with the physiopathology of myelofibrosis itself ( Figure 2). Indeed, there is evidence that myelofibrosis mainly results from a stromal reaction to the clonal hematopoiesis ^62^ as a consequence of the release of profibrotic cytokines ^63, 64^. MKs are the key cells involved in the myelofibrosis because they can release, in the bone marrow, large amounts of profibrotic (transforming growth factor β1 [TGF-β1], basic fibroblast growth factor, and platelet-derived growth factor), angiogenic (vascular endothelial growth factor) and pro-inflammatory (interleukin-1 [IL-1]) cytokines ^62, 65^. The role of MKs in myelofibrosis development explains the link between MK hyperplasia and myelofibrosis. Cytokines such as TGF-β1 are stored in specific MK granules called α-granules. However, in PMF, the most important phenomenon is the MK differentiation defect, which may result in defective α-granule storage and in the release of fibrotic cytokines. It explains why morphological features of MK dysplasia are criteria to distinguish ET from early PMF ^66^. Interestingly, most of the mutations in epigenetic regulators and spliceosome components lead to myeloid differentiation defects, especially in MKs ^67, 68^. Thus, PMF is not a pure MPN, exhibiting myeloproliferative and myelodysplastic features. The heterogeneity of the disease and its prognosis are dependent on the respective levels of each component, and the prognosis is poor when myelodysplastic features are predominant ( Figure 3). This explains why ultimately the prognosis of PMF is mainly dependent on the type and number of mutations in epigenetic regulators and spliceosome genes ^55^. Thus, it is expected that the new entity called pre-PMF or prefibrotic myelofibrosis will have a different pattern of acquired mutations from the classic ET, particularly with the presence of mutations in non-MPN driver genes. Otherwise, factors that regulate MPN phenotype and progression (other than acquired somatic mutations) should be identified.

## Factors other than acquired somatic mutations are involved in the pathogenesis of MPNs

It is clear that factors other than somatic mutations are involved in the pathogenesis of MPNs, particularly in clinical features. They include different factors.

### Germline determinants

Sex-related differences are observed in the distribution of MPNs. ET is predominant in females and PMF in males. There are also differences in the sex ratio between *CALR* mutated and *JAK2*V617F ET. The former are slightly more prevalent in men and the latter in women ^35^. There is no clear explanation for these differences. Hormones could be one explanation. Estrogens can inhibit the *JAK2*V617F cancer stem cells ^69^. Iron metabolism could be another determinant, as it plays an important role in red blood cell and platelet production, with inverse effects.

Other genetic determinants predispose to MPNs. The first characterized was the 46/1 haplotype, which involves the *JAK2* locus ^70–72^. This *JAK2* haplotype induces a 3- to 5-fold increase in *JAK2* V617F MPNs but not in *CALR* mutated MPNs ^73^. Other genetic determinants have recently been found, such as *TERT*, *MECOM*, and *HBS1L/MYB*. The SNPs in *TERT*, *MECOM*, and *JAK2* (other than 46/1) appear to predispose to *JAK2V617F*-negative MPNs, whereas the *HBS1L/MYB* SNPs predispose only to *JAK2V617F* ET ^74^. An SNP located in the *CALR* gene could favor *CALR* mutations ^75^, but this result remains controversial ^76^. It is unknown whether other genetic determinants that regulate blood cell levels regulate the phenotype of MPNs.

The importance of these genetic determinants in the initiation and the progression of MPNs has recently been underscored in four families of the same geographical origin that develop hereditary forms of myeloid malignancies. The transmission is autosomal dominant and leads mainly to ET characterized by the same acquired driver mutations as sporadic cases, but with a very poor prognosis due to a rapid evolution to myelofibrosis and leukemia in more than one third of patients ^77^. A duplication of six genes, two of which are *GSKIP* and *ATG2B*, appears to play a key role in this predisposition, implying that the Wnt pathway and autophagy may play important roles in the pathogenesis of MPNs.

### Inflammation

The JAK-STAT pathway is central for signaling by the majority of the inflammatory cytokines, which were linked to MPN progression. In a study of 30 cytokine levels in 127 patients with PMF, it was found that circulating IL-8, IL-2R, IL-12, and IL-15 levels independently hold prognostic value in PMF ^78^. Overall, many cytokines, including the above markers, G-CSF, and type I interferon (IFN), were increased, whereas IFN-γ was decreased ^78^. Examination of patient-reported outcome and cytokine profiling demonstrated clear associations between MPN symptoms, such as fatigue, abdominal complaints, and microvascular and constitutional symptoms, and high levels of cytokines, particularly IL-1, IL-6, IL-8, and tumor necrosis factor-α (TNF-α) ^79^.

From the pathophysiology standpoint, some pro-inflammatory cytokines or chemokines may be important by directly promoting an extramedullary hematopoiesis. ****In addition, by increasing reactive oxygen species (ROS) production, they may contribute to the dominance of the JAK2V617F clone and disease progression by inducing secondary mutations. A special case is represented by TNF-α. Clonal dominance in JAK2V617F-positive MPNs has been associated with TNF-α secretion and signaling ^80^. TNF-α was also suggested to impair the inhibitory effects of type I IFN on mutated MPN HSCs ^81^. On the other hand, TNF-α inhibition signaling in one patient with myelofibrosis was associated with leukemia progression ^82^. TNF-α also deregulates erythropoietin signaling, leading to anemia in AML and MDS ^83, 84^.

Anemia is also associated with PMF and influences treatment and iron metabolism. Increased levels of both hepcidin and ferritin predicted inferior survival in an independent manner from inflammatory cytokines ^85^.

Co-morbidities can also be coincident with or induced by MPNs. One could ask whether JAK2 inhibitors would impact co-morbidities, which could act on the MPN clone or on the other cells that participate in production and effects of inflammation ^86^. An example is STAT3 activation, which plays an important role in the inflammatory state associated with MPNs. However, when STAT3 is activated in hematopoietic cells from the clone, but not in the other hematopoietic and non-hematopoietic cells, it dampens the MPN phenotype, especially the thrombocytosis ^42^. Chronic inflammation is a driving force for premature atherosclerosis and development of secondary cancer in MPNs ^81^.

### Bone marrow microenvironment: the hematopoietic niche

The anatomical location in which the HSCs reside, the hematopoietic niche, is key for HSC regulation and has been divided into two main compartments: (i) the endosteal niche near the endosteum; and (ii) the perivascular niche near the sinusoids. Many different types of cells compose the niche, mainly derived from mesenchymal stem cells (adipocytes, osteoblasts, and smooth muscle cells) of other origins such as Schwann cells, reticular cells, endothelial cells, and hematopoietic cells such as macrophages, osteoclasts, and MKs. MPN development can be potentially controlled by this bone marrow environment either directly through integrin interactions or indirectly via the production of various chemokines, cytokines, and signaling molecules. Alternatively, mutated HSCs can modify the niche to favor their development and to inhibit normal HSCs to induce clonal expansion. It has been shown that JAK2V617F HSCs secrete IL-1β, which induces the apoptotic death of mesenchymal and Schwann cells, suggesting that the normal but not JAK2V617F HSC is dependent of the niche resulting in a clonal expansion or that JAK2V617F HSCs need to damage the microenvironment to overcome its control. Thus, MPN has been considered a neuropathy that could be controlled by neuroprotective agents ^87^.

In myelofibrosis, the excessive release of fibrotic factors by the mutated MKs could activate mesenchymal cells, leading to myelofibrosis, but also could modify the properties of mesenchymal stromal cells ^88^ and their gene expression ^89^. Some other components of the niche may also belong to the malignant clone. Recently, it has been described that some endothelial cells may also belong to the clone, particularly in the Budd-Chiari syndrome in the liver and the spleen ^90^. Such mutated endothelial cells could potentially be deregulated to exacerbate cytokine or ROS production and to promote platelet adhesion and thrombosis.

Certain cytokines were shown to contribute to MPN development. FLT3L was found to be increased in samples from patients with PMF. It is produced both by HSCs and stromal cells and was shown to participate through the p38 pathway to the dysmegakaryopoiesis and the migration of CD34 ^+^ progenitors ^91^. IL-33 is overproduced in patients with MPN. It contributes to MPN development through stromal cells by promoting cytokine (granulocyte-macrophage colony-stimulating factor and IL-6) secretion via its receptor ST-2 and by amplifying hematopoietic progenitors ^92^.

Nevertheless, the role of the niche in the development of the disease remains incompletely understood. The question of whether an initial abnormality in the bone marrow niche can be the initial event in MPNs remains entirely open. Experimentally, engineered mesenchymal cells could induce hematological malignancies. Deletion of *Dicer1* in mouse osteoprogenitors led to MDS and leukemia through the acquisition of genetic abnormalities ^93^. One of the best ways to study this “niche-induced disease” hypothesis will be to evaluate the role of identified genetic predisposing factors responsible for familial forms of MPNs on the microenvironment and HSCs, respectively, by using engineered mouse models.

### Aging

MPNs are age-related diseases. Both stromal cells and HSCs are modified during aging. With age, HSCs become myeloid-biased with increased cycling/ROS levels and loss of functional capacities that could be important for disease development ^94^. Furthermore, these alterations can eventually favor clonal hematopoiesis with selection of mutated HSCs that acquired independence from stromal regulation. It is noteworthy that the most frequently involved somatic mutations ( *DNMT3A*, *TET2*, *ASXL1*, and *JAK2*) linked to aging are also implicated in myeloid malignant hematological malignancies, including MPNs.

## Conclusion

The understanding of the MPN pathogenesis, including ET and PMF, has greatly progressed these 10 last years because of the discovery of the main MPN driver mutations. More than 90% of non- *BCR-ABL* MPNs are clearly driven by an abnormal JAK2 activation, especially the cytokine receptor/JAK2 pathways and their downstream effectors. Genomic studies demonstrated that PMF is a more advanced form of MPN, but with a molecular redundancy with ET. However, in contrast to classic ET and PV, PMF constantly includes one or several mutations in non-MPN driver genes, which are present also in MDS. This and the cytological features of the disease strongly suggest that PMF is a heterogeneous disorder associating phenotype/genotype features of MPN and MDS, with the latter being crucial for prognosis.

Several important questions remain to be solved:

-   What are the mechanisms of disease initiation? Indeed, *JAK2*V617F can be frequently acquired but rarely gives rise to a disease.
-   Why in ET and PMF do JAK2V617F and mutant CALR pathways give rise to close but different diseases, while they both activate MPL/JAK2? A similar question may arise for type 1 and type 2 CALR mutations.
-   Why can a mutation like *JAK2*V617F give rise to several diseases?
-   What are the molecular mechanisms of oncogenic cooperation between MPN driver mutations and other acquired somatic mutations? How does this oncogenic cooperation lead to leukemia?

In all cases, one major question remains to be solved: what are the respective roles of the genetic abnormalities, either germline or acquired (intrinsic factors), and of the environment (extrinsic factors) in disease initiation, phenotype, and progression?

Finally, from the therapeutic point of view, new approaches which will preferentially target an oncogenic JAK2 activation versus the physiological JAK2 role in cytokine signaling remain to be identified. In PMF with a high level of myelodysplastic features, this type of approach might not be sufficient and will require novel combined approaches.

## Abbreviations

AML, acute myeloid leukemia; CALR, calreticulin; ER, endoplasmic reticulum; ET, essential thrombocythemia; G-CSF, granulocyte colony-stimulating factor; HSC, hematopoietic stem cell; IFN, interferon; IL, interleukin; MDS, myelodysplastic syndrome; MK, megakaryocyte; MPN, myeloproliferative neoplasm; NGS, next-generation sequencing; PMF, primary myelofibrosis; PV, polycythemia vera; ROS, reactive oxygen species; TGF-β1, transforming growth factor β1; TNF-α, tumor necrosis factor-α.
